# Highly Effective Markerless Genetic Manipulation of *Streptococcus suis* Using a Mutated PheS-Based Counterselectable Marker

**DOI:** 10.3389/fmicb.2022.947821

**Published:** 2022-07-14

**Authors:** Guangjuan Gao, Dong Wei, Gang Li, Ping Chen, Liujun Wu, Siguo Liu, Yueling Zhang

**Affiliations:** State Key Laboratory of Veterinary Biotechnology, Harbin Veterinary Research Institute, Chinese Academy of Agricultural Sciences, Harbin, China

**Keywords:** *Streptococcus suis*, markerless genetic manipulation, mutated PheS, counterselectable marker, strong promoter

## Abstract

*Streptococcus suis* is an important zoonotic pathogen, however, an efficient markerless genetic manipulation system is still lacking for further physiological and pathological studies on this bacterium. Several techniques have been developed for markerless genetic manipulation of *S. suis* utilizing either a temperature-sensitive vector or a counterselectable markers (CSMs), however, at present, the efficiency of these techniques is not very satisfactory. In this study, we developed a strategy for markerless genetic manipulation of *S. suis* employing a CSM based on a conditionally lethal mutant allele of *pheS*, which encodes the α-subunit of phenylalanyl-tRNA synthetase (PheS). This mutant *pheS*, *mPheS*, was constructed by introducing site-directed mutations for a T261S/A315G double-substitution and a number of silent mutations to decrease its similarity with the endogenous wild type *pheS* gene (*wtPheS*). Additionally, five potentially strong promoters from *S. suis* were screened for their ability to drive high-level expression of mPheS, thus endowing the carrier strain with sufficient sensitivity to the phenylalanine analog *p*-chloro-phenylalanine (*p*-Cl-phe). Insertion of these P-*mPheS* cassettes into a vector or into the chromosomal locus *via* a linked erythromycin resistance gene revealed that *mPheS* allele driven by promoters P_0530_ and P_1503_ renders *S. suis* sensitive to as low as 0.01% (or 0.5 mM) of *p*-Cl-phe. This offers two potential CSMs for *S. suis* with *p*-Cl-phe as a counterselective agent. P_1503_-*mPheS* was revealed to be 100% efficient for counter-selection in *S. suis* by application in a precise gene deletion. Using P_1503_-*mPheS* as a CSM, a two-step insertion and excision strategy for markerless genetic manipulation of *S. suis* were developed, supplying a powerful tool for markerless genetic manipulation of *S. suis*.

## Introduction

*Streptococcus suis* is a Gram-positive bacterium and is associated with a wide range of diseases in pigs, including meningitis, arthritis, septicemia, and death (Segura, 2009; Palmieri et al., 2012; Goyette-Desjardins et al., 2014). More seriously, it can be transmitted to humans by exposure to sick pigs, causing meningitis, streptococcal toxic shock-like syndrome, and death. *S. suis* was reported to be an emerging and re-emerging zoonotic pathogen, and seriously threatens the swine industry and human public health (Lun et al., 2007; Palmieri et al., 2012; Feng et al., 2014; Goyette-Desjardins et al., 2014). During the last two decades, *S. suis* has attracted widespread attention from researchers, and significant progress has been made in understanding its physiological and pathological process (Segura et al., 2017; Lin et al., 2019; Tan et al., 2019, 2021; Xie et al., 2019; Chen et al., 2020). However, further sophisticated and unbiased investigation of *S. suis*’s physiology and pathology process urgently needs an efficient genetic manipulation system for precise and clean genetic manipulation such as scarless gene-deletion, gene-fusion, and point-mutation.

In *S. suis*, the most frequently used genetic manipulation system is based on the pSET4s thermosensitive suicide vector (Takamatsu et al., 2001b). The procedure involved the introduction of vector into *S. suis* by electroporation, and two steps of allelic exchange. However, this system is labor-intensive and time-consuming, and electrotransformation does not work well for certain *S. suis* isolates. Recently, a cloning-independent method employing peptide-induced competence has been established in *S. suis* (Zaccaria et al., 2014). It allows high-throughput mutation, but the resulted mutant must carry an antibiotic-resistance gene for selection, limiting its use in markerless and multiple gene manipulations.

Counterselection systems have been reported as an efficient way to establish markerless mutants in many bacteria (Kristich et al., 2005; Kino et al., 2016; Argov et al., 2017). Only very recently, counterselectable markers (CSMs) were inspected for their use in *S. suis* genetic manipulation. The well-known *Bacillus subtilis* levansucrase SacB and the *Vibrio parahaemolyticus* toxin YoeB have been tested for their potential for counter-selection in *S. suis* (Zhang et al., 2019; Zheng et al., 2021). Though the target markerless mutations have been successfully selected by these CSMs, the notorious counter-selection escape of the SacB-based system and the incomplete growth inhibition of the YoeB-based system strongly limited the efficiency of these systems and urged us to explore a more effective CSM alternative for *S. suis*.

Among the known counterselectable genes, derivatives of the *pheS* gene, which encodes the α-subunit of phenylalanyl-tRNA ligase PheS, has recently been reported to be an effective CSM for genetic deletion in several bacteria (Kino et al., 2016; Zhou et al., 2017; Kharchenko et al., 2018; Liu et al., 2018, 2020; Wang et al., 2018; Schuster et al., 2019). The *Escherichia coli* PheS mutant, containing an A294G single-substitution, is able to misincorporate the phenylalanine analog *p*-chloro-phenylalanine (*p*-Cl-phe) into proteins during translation, thereby causing cell death (Kast and Hennecke, 1991). After the initial use for counter-selection in *E. coli*, various *pheS* markers were then adapted for the genetic engineering of both Gram-negative and Gram-positive bacteria (Kast, 1994; Kristich et al., 2007; Argov et al., 2017; Zhou et al., 2017). The drawback of this single-substitution mutant is its rather low selection efficiency, which requires the selection to be performed on a minimal or semi-rich medium with relatively high concentrations of *p*-Cl-phe (>5 mM). Later Miyazaki (2015) demonstrated that the T251S/A294G double-substituted PheS mutant possessed higher *p*-Cl-phe incorporation efficiency, and Kharchenko et al. (2018) proved that in *Bacillus* this double-substituted PheS mutant confers higher sensitivity to *p*-Cl-phe even in rich medium.

In order to develop an effective CSM, and establish a markerless genetic manipulation strategy for *S. suis*, in this study, a synthetic *pheS* gene mutant (*mPheS*) was designed with two site-directed mutations and a number of silent mutations from the native wild type *pheS* gene (*wtPheS*). Driven by a strong promoter, this *mPheS* enables an extremely efficient two-step strategy for markerless genetic manipulation of *S. suis*.

## Materials and Methods

### Bacterial Strains, Plasmids, and Growth Conditions

*Streptococcus suis* strain 05ZYH33 was used as a wild type strain (WT) (Chen et al., 2007). *S. suis* strains were cultured at 37°C with 5% CO_2_ on Tryptic Soy Agar (TSA) or in Tryptic Soy broth (TSB) supplemented with 5% (vol/vol) horse serum (Biosharp). *E. coli* strains were grown in Luria–Bertani (LB) broth or on LB agar at 37°C. All pSET2-derived plasmids were propagated in *E. coli* MC1061F-strain (Takamatsu et al., 2001a). When required, the indicated concentration of *p*-Cl-phe (Sigma) was added to the TSA before autoclaving. Spectinomycin was added to the medium at 50 μg/ml for *E. coli* and 100 μg/ml for *S. suis*. Erythromycin was added to the medium at 4 μg/ml for *S. suis*.

### Design and Synthesis of *pheS* Gene Mutant

Wild type *pheS* gene was searched from the genome of *S. suis* 05ZYH33 (GenBank: CP000407) by BLAST with the *pheS* of *S. mutans* (Zhang et al., 2017). *mPheS* was then designed based on *wtPheS* nucleotide sequence by introducing a double-substitution and a number of silent mutations. To introduce the double-substitution corresponding to T251S/A294G of *E. coli* PheS, the threonine and alanine counterparts were identified by sequence alignment of PheS proteins from *E. coli*, *S. mutans*, *Listeria monocytogenes*, *Bacillus amyloliquefaciens*, and *S. suis* using ClustalW. To introduce silent mutations, codon adaptation software Jcat (Grote et al., 2005) was employed to perform one round of codon adaption using *S. pyogenes* codon usage as the reference, then manual modification was applied to further decrease the nucleotide sequence similarity between *mPheS* to *wtPheS*. The final modified *mPheS* gene was synthesized by BGI (Beijing, China).

### Construction of Plasmid-Carried *mPheS* Driven by Different Promoters

Five potentially strong promoters P_0177_, P_0530_, P_1503_, P_1815_, and P_1868_ were used to drive the expression of *mPheS*, in comparison with the native *pheS* promoter (P_*wt*_). These five promoters have previously been identified as strong promoters for two reasons. First, they are responsible for the expression of five high abundant proteins in *S. suis* 05ZYH33, with genome locus_tag of SSU05_0177, SSU05_0530, SSU05_1503, SSU05_1815, and SSU05_1868, respectively. Second, they could drive high level expression of exogenous GFP in *S. suis* (Liu et al., 2019). In order to construct the P-*mPheS* cassettes (*mPheS* gene driven by different promoters), *mPheS* gene was amplified from the above synthesized *mPheS* gene with primers mPheS-F/mPheS-R. Six promoters, including the five potentially strong promoters and P_*wt*_ were amplified from *S. suis* 05ZYH33 chromosomal DNA using corresponding primers listed in Table 1. The fragment P_*wt*_-wtPheS which contains native *pheS* gene and its promoter was also amplified to be used as a control. The P_*wt*_-*mPheS*, P_0177_-*mPheS*, P_0530_-*mPheS*, P_1503_-*mPheS*, P_1815_-*mPheS*, and P_1868_-*mPheS* cassettes were constructed using overlap-extension PCR strategy, with the amplicons of *mPheS* and each of the six promoters as templates. These cassettes and P_*wt*_-wtPheS were recombinationally cloned into *Eco*RI/*Bam*HI double-digested shuttle vector pSET2 using Trelief SoSoo cloning Kit Ver. 2 (Tsingke, Beijing), and verified by PCR and sequencing with primers pSET2-F/pSET2-R. All the primers used in this study are listed in Table 1.

**TABLE 1:** Primers used in this study.

Name	Sequence (5′–3′)	Size (bp)
**For construction of plasmid containing P-*mPheS* cassette**		
pP_*wt*_-wtPheS-F	ACGACGGCCAGTGAATTCACAGTCAGTATTCCCTCA	1224
pP_*wt*_-wtPheS-R	AGGTCGACTCTAGAGGATCCTCAAAACTGCTCCGAGA	
pP_0177_-F	ACGACGGCCAGTGAATTCTTGGTAAGAGAAATGTGAGTG	484
pP_0177_-R	**GTTGTTGCTCGATGTTAGACAT**ATCTTTATAAGACATGATATCCTC	
pP_0530_-F	ACGACGGCCAGTGAATTCGTAGGATAACTGAATGGAGAA	300
pP_0530_-R	**GTTGTTGCTCGATGTTAGACAT**TTTGGTAAAAGCCTCCAATAA	
pP_1503_-F	ACGACGGCCAGTGAATTCTGTTTCGCCAGAGGCTT	197
pP_1503_-R	**GTTGTTGCTCGATGTTAGACAT**TATATTACTCTCCTTTGAGTTT	
pP_1815_-F	ACGACGGCCAGTGAATTCCAGCGCCTCAAAAACTA	352
pP_1815_-R	**GTTGTTGCTCGATGTTAGACAT**AAGTCCTCCATATAAGTACTTC	
pP_1868_-F	ACGACGGCCAGTGAATTCAAAAACAGCAAGGATTGTAG	257
pP_1868_-R	**GTTGTTGCTCGATGTTAGACAT**AAAACACCTCTGTTTTCTTT	
pP_*wt*_-F	ACGACGGCCAGTGAATTCTAATTGAATAGAAGTCTGTGAGAC	300
pP_*wt*_-R	**GTTGTTGCTCGATGTTAGACAT**AATTCCTCCAATAAAAAACGC	
mPheS-F	ATGTCTAACATCGAGCAAC	1044
mPheS-R	AGGTCGACTCTAGAGGATCCTTAGAATTGTTCTGAGAAACGAAC	
pSET2-F	AACTGTTGGGAAGGGCGA	
pSET2-R	GTGGAATTGTGAGCGGATAA	
**For construction of strains with genome-integrated P-*mPheS-erm* cassette**		
UP_0630_-F	TGCTAACGATGCTACAAATGC	1024
UP_0630_-R	TTACTCCTTCTTCCGCCGG	
gP_0177_-PheS-F	**CCGGCGGAAGAAGGAGTAA**TTGGTAAGAGAAATGTGAGTG	1528
gP_0530_-PheS-F	**CCGGCGGAAGAAGGAGTAA**GTAGGATAACTGAATGGAGAA	1344
gP_1503_-PheS-F	**CCGGCGGAAGAAGGAGTAA**TGTTTCGCCAGAGGCTT	1241
gP_1815_-PheS-F	**CCGGCGGAAGAAGGAGTAA**CAGCGCCTCAAAAACTA	1396
gP_1868_-PheS-F	**CCGGCGGAAGAAGGAGTAA**AAAAACAGCAAGGATTGTAG	1301
gP_*wt*_-PheS-F	**CCGGCGGAAGAAGGAGTAA**TAATTGAATAGAAGTCTGTGAGAC	1344
gP-PheS-R	TTAGAATTGTTCTGAGAAACGAACG	
Erm-DN_0630_-F	**CGTTCGTTTCTCAGAACAATTCTAA**AGAAGGAGGGATTCGTCATG	2413
Erm-DN_0630_-R	CAAAGATAGCGGTGGTCGT	
SeqF	GCGGAGCCCTTACCAG	
SeqR	AATACAGAAGTTAAACGATTTGT	
**For construction of *ireB* markerless gene-deletion strain**		
UP1-F	GAAGAAGCTCCTGTTGTTGC	827
UP1-R	CTTCGGTAAATCCCATACTTAC	
PPE-F	**GTAAGTATGGGATTTACCGAAG**TGTTTCGCCAGAGGCTT	2435
PPE-R	**GTCAATCCCATTCCCTTTC**CCAAATTCCCCGTAGGC	
DN1-F	GAAAGGGAATGGGATTGAC	718
DN1-R	GCGTCTTCTGGGATAGGTT	
UP2-F	ACAACGCCTGGTGGACG	1209
UP2-R	ACTTACACCTTCTTTCCCT	
DN2-F	**AGGGAAAGAAGGTGTAAGT**TGAGAATAATGGGATTAGACGT	1228
DN2-R	TGATAGGCTGGATAGTTTTGATA	
ireBseq-F	ACGCAGTAGCTCAAGCC	
ireBseq-R	ATTCCATAACATAATCTCCC	
ireB-F	GAAACGACTTCAAGTGGGC	
ireB-R	GTTCGGTCAAACGCTCCA	

*Underlined indicates the recombination sequences introduced for cloning into pSET2. Bold indicates the overlapped sequences used for overlap-extension PCR.*

### Integrating P-*mPheS* Cassettes Into *Streptococcus suis* Genome *via* a Linked Erythromycin Resistance Gene

To integrate the P-*mPheS* cassettes into the genome of *S. suis*, an appropriate integration site, and a linked positive-selection marker are needed. In this study, the site right downstream of gene *ssu05-0630* was chosen as the integration site on the basis of our previous construction of an erythromycin-resistant-gene (*erm*) substituted *ssu05-0630* gene-deletion strain (Chen et al., 2019, 2020). It has been shown that the gene modification at this site does not affect the growth of *S. suis* (Chen et al., 2019). Furthermore, the positive-selection marker *erm* and the downstream sequence can be conveniently amplified as one amplicon from the gene-deletion strain. To construct the fused DNA fragments for integration, the upstream sequence (UP_0630_) and the *erm* marker with the downstream sequence (Erm-DN_0630_) were amplified from the *ssu05-0630* gene-deletion strain. The six P-*mPheS* cassettes were amplified from the above constructed plasmids with the corresponding primers listed in Table 1. Each of the P-*mPheS* cassettes was fused with UP_0630_ and *erm*-DN_0630_ using overlap-extension PCR in the order of UP_0630_-P-*mPheS*-*erm*-DN_0630_. The fused DNA fragments were transformed into *S. suis* 05ZYH33, and plated on TSAS-Erm medium (TSA supplemented with horse serum and erythromycin) for positive-selection. The integration of P-*mPheS* cassettes in positive clones was confirmed by PCR and sequencing using primers SeqF/SeqR.

### Peptide-Induced Transformation

A peptide-induced transformation was performed as previously described (Zaccaria et al., 2014) with slight modifications. The peptide (GNWGTWVEE) was synthesized by GenScript (China) at 95% purity. It was dissolved in deionized water at a final concentration of 5 mM, divided into aliquots, and stored at −20°C. Overnight culture of *S. suis* 05ZYH33 was diluted 1:100 in fresh TSBS medium and grown for 1.5, 2, 2.5, and 3 h. For each time point, a 50 μl culture was collected, and mixed with 2.5 μl of peptide and 1 μg of DNA (plasmid or PCR product). Following 4 h of incubation, the mixtures were plated on TSAS (TSA supplemented with horse serum) containing spectinomycin, erythromycin, or *p*-Cl-phe for selection.

### Growth Inhibition of *p*-Chloro-Phenylalanine to *Streptococcus suis* Strains

To inspect the sensitivity of *S. suis* strains to *p*-Cl-phe, growth inhibition test was performed. Overnight cultures of *S. suis* strains were diluted in fresh medium and grown to OD_600__*nm*_ 0.6. Each culture was undiluted or 10-fold serially diluted up to 10^–5^, and 5 μl of each dilution was spotted on to TSAS plate supplemented with indicated concentrations of *p*-Cl-phe. The growth of strains on the plates was photographically documented after 24 h incubation at 37°C, 5% CO_2_. The minimum inhibitory concentration (MIC) for each strain was defined as the lowest concentration of *p*-Cl-phe that inhibits the visible growth of that strain.

### Construction of a *Streptococcus suis* Markerless Gene-Deletion Mutant Using the Established Counterselectable Marker

To test the efficiency of the P_1503_-*mPheS* as a CSM, it was used for markerless deletion of *ireB* gene (genome locus_tag SSU05_0066). It is a homolog of the *ireB* gene from *Enterococcus faecalis* and *reoM* gene from *L. monocytogenes*, both were reported to play important roles in peptidoglycan synthesis (Hall et al., 2013; Wamp et al., 2020). The whole process involved the construction of two fused DNA fragments and twice transformation. Using the corresponding primers listed in Table 1 for *ireB* gene-deletion, the upstream and downstream sequences, i.e., UP1, DN1, UP2, DN2, of *ireB* gene were amplified from the WT strain, and the P_1503_-*mPheS*-*erm* (designated as P_1503_PE) sequence was amplified from the strain with genome-integrated P_1503_PE cassette. For the first transformation, the first fused fragment of P_1503_PE flanked by UP1 and DN1 was prepared by overlap-extension PCR. The resulted UP1-P_1503_PE-DN1 fragment was transformed into WT and screened on TSAS-Erm plates. Positives colonies were inoculated on TSAS plate supplemented with 0.05% *p*-Cl-phe to confirm its *p*-Cl-phe sensitivity, yielding the P_1503_PE-substituted *ireB* gene-deletion intermediate strain, *ireB*^Δ^PPE. For the second transformation, the second UP2-DN2 fusion fragment was prepared and transformed into the above *p*-Cl-phe-sensitive *ireB*^Δ^PPE intermediate strain, and counter-selected on the TSAS plate supplemented with 0.05% *p*-Cl-phe. The positive clones were verified by PCR with primers ireB-F/ireB-R. The percentage of *ireB* gene-deleted colonies was determined by random analysis of 100 colonies. The experiment of *ireB* gene-deletion was performed three times to confirm the counter-selection efficiency.

### Nucleotide Sequence Accession Number

The DNA sequence of the modified *mPheS* gene has been deposited in GenBank under the accession number: ON184273.

## Results

### The *Streptococcus suis* Endogenous *wtPheS* and Its *mPheS* Derivate

**FIGURE 1:**
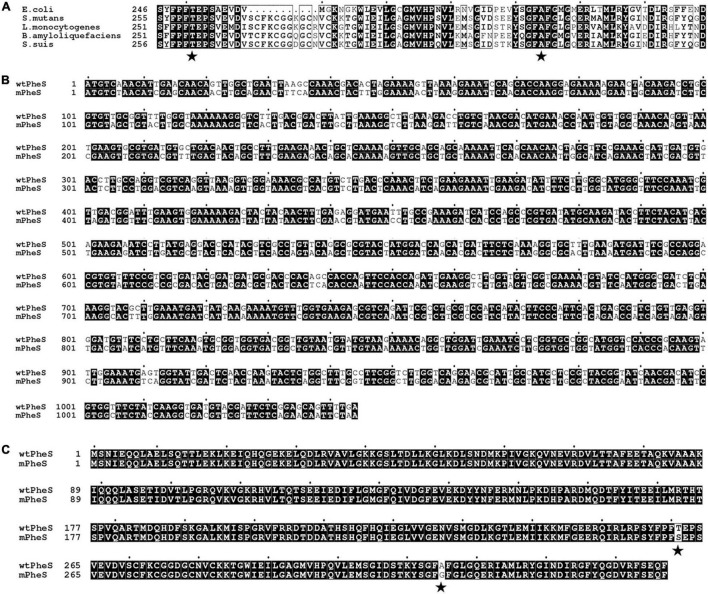
PheS-related sequence alignment. **(A)** Amino acid sequence alignment of PheS proteins from *E. coli*, *S. mutans*, *Listeria monocytogenes*, *Bacillus amyloliquefaciens*, and *S. suis*. The T251 and A294 of PheS from *E. coli* and corresponding counterparts were indicated by stars. **(B)** Nucleotide sequence alignment of the *wtPheS* and *mPheS* genes. **(C)** Amino acid sequence alignment of the wtPheS and mPheS proteins. The T261S and A315G substitutions were indicated by stars.

The wtPheS with locus_tag of SSU05_1152 was identified from *S. suis* 05ZYH33 genome. Sequence alignment revealed that T261 and A315 are the counterparts corresponding to the T251 and A294 of *E. coli* PheS protein (Figure 1A). To develop a mPheS-based CSM, functional similarity is important for competition with the endogenous wtPheS for PheT-PheS complex formation (Ibba et al., 1994; Argov et al., 2017), therefore, a *mPheS* gene mutant was directly derived from the *wtPheS* based on the following two considerations. First, to effectively misincorporate *p*-Cl-phe, T261 and A315 were substituted by serine and glycine, respectively. Second, in order to avoid unwanted homologous recombination, silent mutations were introduced into *mPheS* to decrease the nucleotide sequence homology between the *wtPheS* and *mPheS* allele. As shown in Figures 1A,B, after double-substitution, codon adaption and manual modification, the resulting *mPheS* showed 73.7% similarity to *wtPheS* gene, and the longest continuously matched sequence between them is no more than 8 bp. Meanwhile, the protein sequence encoded by the *mPheS* showed exact identity with the wtPheS protein, except for the T261S and A315G double-substitution (Figure 1C).

### P_0530_-*mPheS* and P_1503_-*mPheS* Cassettes Have Great Potential as Counterselectable Marker for *Streptococcus suis* When Carried by the Plasmid

**FIGURE 2:**
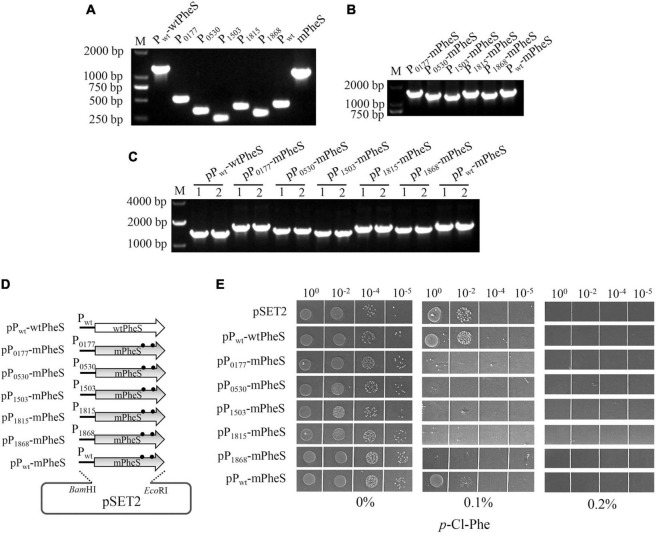
Construction and growth inhibition test of *S. suis* strains with plasmid-carried P-*mPheS* cassettes. **(A)** Amplification of P_*wt*_-*wtPheS*, the six promoters (P_0177_, P_0530_, P_1503_, P_1815_, P_1868_, P_*wt*_) and *mPheS* fragments. **(B)** Fused P-*mPheS* cassettes. **(C)** PCR confirmation of the *S. suis* transformants containing plasmid-carried P-*mPheS* cassettes (pP-*mPheS*) with primers pSET2-F/pSET2-R. **(D)** Diagram for the constructed plasmids carrying P-*mPheS* cassettes. **(E)** Growth inhibition of pP-*mPheS* strains by the indicated concentrations of *p*-Cl-phe.

To explore whether *mPheS* has potential as a CSM for *S. suis*, shuttle vector pSET2 was used to carry *mPheS* gene into *S. suis*. Furthermore, it was reported that high level of mPheS expression is important for functional competition with the endogenous wtPheS, which is key for effective misincorporation of *p*-Cl-phe and growth inhibition (Argov et al., 2017). To achieve high-level expression of *mPheS* allele, five previously identified potentially strong promoters (P_0177_, P_0530_, P_1503_, P_1815_, and P_1868_) (Liu et al., 2019) were selected to drive the gene expression of *mPheS* in addition to the native promoter P_*wt*_. As shown in Figure 2, to construct the *S. suis* strains with pSET2-carried P-*mPheS* cassettes, fragments of P_*wt*_-*wtPheS*, P_0177_, P_0530_, P_1503_, P_1815_, P_1868_, P_*wt*_, and *mPheS* were amplified accordingly (Figure 2A). Then the six promoters were individually fused with *mPheS* gene, producing P_0177_-*mPheS*, P_0530_-*mPheS*, P_1503_-*mPheS*, P_1815_-*mPheS*, P_1868_-*mPheS*, and P_*wt*_-*mPheS* cassettes (Figure 2B). P_*wt*_-wtPheS and the six P-*mPheS* cassettes were recombinationally inserted into pSET2, and transformed into WT, yielding strains containing pSET2-carried *mPheS* driven by different promoters, namely pP_*wt*_-*wtPheS*, pP_0177_-*mPheS*, pP_0530_-*mPheS*, pP_1503_-*mPheS*, pP_1815_-*mPheS*, pP_1868_-*mPheS*, and pP_*wt*_-*mPheS* (Figures 2C,D and Table 2).

**TABLE 2:** Strains constructed in this study.

Name	Description
pP_*wt*_-*wtPheS*	*S. suis* 05ZYH33 containing pSET2-carried P_*wt*_-wtPheS cassette
pP_0177_-*mPheS*	*S. suis* 05ZYH33 containing pSET2-carried P_0177_-mPheS cassette
pP_0530_-*mPheS*	*S. suis* 05ZYH33 containing pSET2-carried P_0530_-mPheS cassette
pP_1503_-*mPheS*	*S. suis* 05ZYH33 containing pSET2-carried P_1503_-mPheS cassette
pP_1815_-*mPheS*	*S. suis* 05ZYH33 containing pSET2-carried P_1815_-mPheS cassette
pP_1868_-*mPheS*	*S. suis* 05ZYH33 containing pSET2-carried P_1868_-mPheS cassette
pP_*wt*_-*mPheS*	*S. suis* 05ZYH33 containing pSET2-carried P_*wt*_-mPheS cassette
gP_0177_PE	*S. suis* 05ZYH33 with genome-integrated P_0177_-*mPheS-erm* cassette
gP_0530_PE	*S. suis* 05ZYH33 with genome-integrated P_0530_-*mPheS-erm* cassette
gP_1503_PE	*S. suis* 05ZYH33 with genome-integrated P_1503_-*mPheS-erm* cassette
gP_1815_PE	*S. suis* 05ZYH33 with genome-integrated P_1815_-*mPheS-erm* cassette
gP_1868_PE	*S. suis* 05ZYH33 with genome-integrated P_1868_-*mPheS-erm* cassette
*ireB*^Δ^ PPE	*S. suis* 05ZYH33 intermediate mutant with the *ireB* gene substituted by P_1503_PE
*ireB* ^Δ^	*S. suis* 05ZYH33 markerless *ireB* gene-deletion mutant

Next, the *p*-Cl-phe sensitivity endowed by P-*mPheS* cassettes to the carrier strains was inspected. Growth inhibition by *p*-Cl-phe was tested with TSAS plates containing 0, 0.1, and 0.2% *p*-Cl-phe. Strains containing pP_*wt*_-*wtPheS* and empty pSET2 were both used as control that expresses wtPheS but not mPheS. As shown in Figure 2D, all the strains grew well on the TSAS without *p*-Cl-phe. No growth was observed with 0.2% *p*-Cl-phe, even for the control strains without mPheS expression, indicating that 0.2% *p*-Cl-phe was too high for *S. suis* counter-selection. With 0.1% *p*-Cl-phe, clear differences in sensitivity were displayed by strains carrying different P-*mPheS* cassettes. Only slight growth inhibition was observed for control strains expressing no mPheS, as well as the strain carrying *mPheS* driven by P_*wt*_ (pP_*wt*_-mPheS). The latter indicates that mPheS protein could not compete well with the endogenous wtPheS protein when they have similar expression levels. In contrast, the growth of the five strains containing *mPheS* driven by potentially strong promoters was significantly inhibited. No growth was observed even for the un-diluted culture of strains containing P_0530_-*mPheS* and P_1503_-*mPheS* cassettes, while only a few colonies were observed for the un-diluted culture of strains containing P_0177_-*mPheS*, P_1815_-*mPheS*, and P_1868_-*mPheS* cassettes. This indicates that mPheS expression driven by all these five promoters is able to overwhelmingly compete with the endogenous wtPheS, thus endowing the carrier strains with sufficient sensitivity to *p*-Cl-phe. Therefore, P_0177_-*mPheS*, P_0530_-*mPheS*, P_1503_-*mPheS*, P_1815_-*mPheS*, and P_1868_-*mPheS* cassettes all have potential as a CSM for *S. suis*, among them P_0530_-*mPheS* and P_1503_-*mPheS* have the greatest potential.

### P_0530_-*mPheS* and P_1503_-*mPheS* Cassettes Have Great Potential as Counterselectable Marker for *Streptococcus suis* When Integrated Into Genome

**FIGURE 3:**
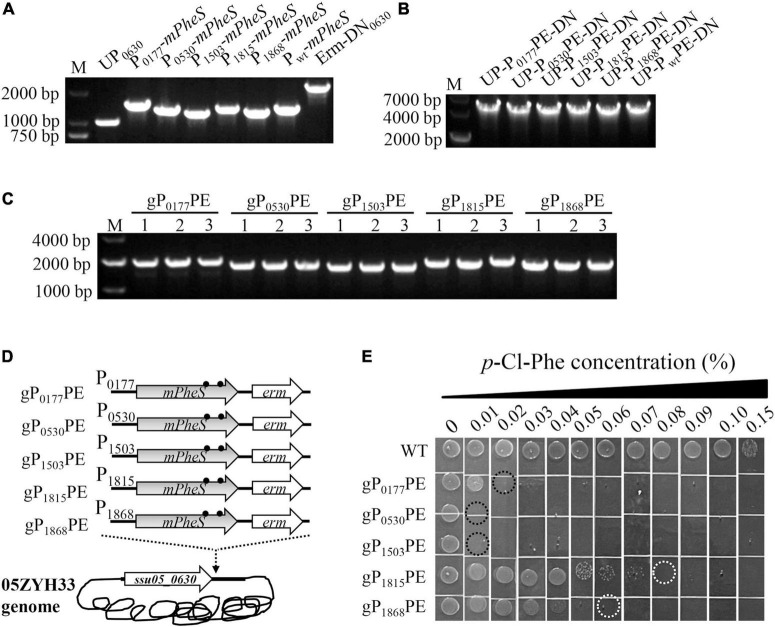
Construction and growth inhibition test of *S. suis* strains with genome-integrated P-*mPheS* cassettes. **(A)** Amplification of UP_0630_, the six P-*mPheS* cassettes, and Erm-DN_0630_ fragments. **(B)** Fused UP-PPE-DN fragments. **(C)** PCR confirmation of *S. suis* transformants with genome-integrated P-*mPheS* cassettes (gPPE) with primers SeqF/SeqR. **(D)** Diagram for the constructed *S. suis* with genome-integrated P-*mPheS* cassettes. **(E)** Growth inhibition of gPPE strains by the indicated concentrations of *p*-Cl-phe. The MIC for each strain was indicated with dashed cycle.

To further test the potential of the P-*mPheS* cassettes as CSM for *S. suis* when they were integrated into the genome *via* a positive-selection marker, which is the real case for counter-selection, they were integrated into the site right downstream of *ssu05_0630* gene *via* an *erm* marker. As shown in Figure 3, the upstream sequence (UP_0630_) and the *erm* marker with downstream sequence (Erm-DN_0630_) were amplified from the *erm*-substituted *ssu05-0630* gene-deletion strain we previously constructed, while the six P-*mPheS* cassettes (including the P_*wt*_-*mPheS* cassette) were amplified from the above-constructed plasmids (Figure 3A). Each of the six P-*mPheS* fused with UP_0630_ and *erm*-DN_0630_, yielding fused DNA fragments of UP-P_0177_PE-DN, UP-P_0530_PE-DN, UP-P_1503_PE-DN, UP-P_1815_PE-DN, UP-P_1868_PE-DN, and UP-P_*wt*_PE-DN (for short, P-*mPheS*-*erm* was designated as PPE) (Figure 3B). After transformation, it was confirmed that the cassettes P_0177_PE, P_0530_PE, P_1503_PE, P_1815_PE, and P_1868_PE were successfully integrated into the site right downstream of *ssu05-0630* gene (Figure 3C), yielding strains gP_0177_PE, gP_0530_PE, gP_1503_PE, gP_1815_PE, and gP_1868_PE (Figure 3D and Table 2). However, no transformant was obtained for the UP-P_*wt*_PE-DN fragment, very possibly due to the high sequence identity to the endogenous P_*wt*_-*wtPheS* locus, leading to off-target recombination and failure of targeted integration.

The *p*-Cl-phe sensitivity of these strains was tested with TSAS plates containing 0–0.15% *p*-Cl-phe. As shown in Figure 3E, the WT strain grew well with *p*-Cl-phe concentrations below 0.1%, with the MIC of *p*-Cl-phe above 0.15%. In contrast, all five strains integrated with PPE were sensitive to 0.1% of *p*-Cl-phe. The MIC of *p*-Cl-phe for strains gP_0177_PE, gP_0530_PE, gP_1503_PE, gP_1815_PE, and gP_1868_PE were 0.02, 0.01, 0.01, 0.08, and 0.06%, respectively (Figure 3E). These results are in good consistent with those obtained with the above pP-*mPheS* strains, further confirming that *mPheS* driven by all these five promoters has potential as a CSM for *S. suis* counter-selection. Furthermore, as the gP_0530_PE and gP_1503_PE strains have the lowest MIC of *p*-Cl-phe, which is as low as 0.01%, less than one fifteenth of the MIC for the WT strain, the P_0530_-*mPheS* and P_1503_-*mPheS* cassettes in these strains have the greatest potential as a CSM for *S. suis*. Considering the promoter is an endogenous sequence from *S. suis*, the shorter, the less possibility of unwanted recombination. Therefore, the P_1503_-*mPheS* cassette containing the shorter promoter (197 bp of P_1503_ vs. 530 bp of P_0530_) suits better as a CSM for *S. suis*, but P_0530_-*mPheS* cassette still can be a good option when needed.

### P_1503_-*mPheS* Cassette Is an Extremely Efficient Counterselectable Marker for *Streptococcus suis*

**FIGURE 4:**
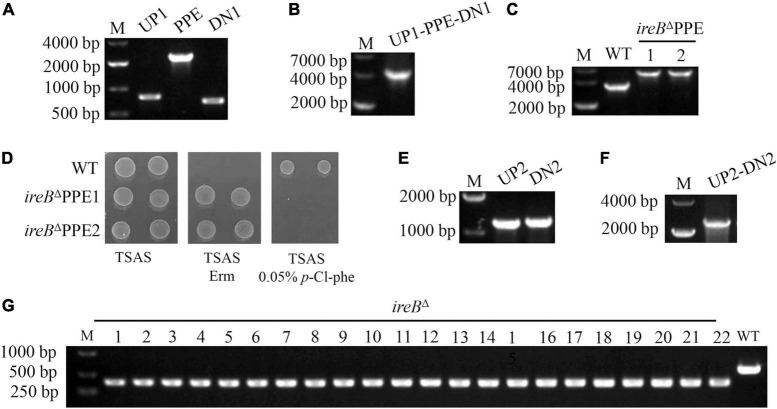
Markerless deletion of *ireB* gene using the P_1503_-*mPheS* cassette as a CSM. **(A)** Amplification of upstream sequence (UP1) and downstream sequence (DN1) of *ireB* gene and P_1503_PE cassette. **(B)** Fused UP1-P_1503_PE-DN1 fragment. **(C)** PCR confirmation of two *ireB*^Δ^ PPE intermediate transformants with primers ireBseq-F/ireBseq-R. **(D)** Confirmation of the resistance to erythromycin and the sensitivity to 0.05% *p*-Cl-phe for two *ireB*^Δ^ PPE intermediate strains (*ireB*^Δ^ PPE1, *ireB*^Δ^ PPE2). **(E)** Amplification of upstream sequence (UP2) and downstream sequence (DN2) of *ireB* gene. **(F)** Fused UP2-DN2 fragment. **(G)** PCR confirmation of the colonies survived from counter-selection with primers ireB-F/ireB-R.

To evaluate the efficiency of P_1503_-*mPheS* cassette as a CSM for *S. suis*, we applied it in the construction of a markerless *ireB* gene-deletion mutant. We choose this gene because when we tried to investigate the function of IreB protein in *S. suis*, we found that *ireB* gene locates in an operon containing 4–5 genes (data not shown), therefore, markerless deletion of *ireB* gene is necessary to avoid the unwanted polar effect. First, the upstream and downstream sequences of *ireB* gene, i.e., UP1 and DN1 and the P_1503_PE fragment was amplified from WT and gP_1503_PE strain, respectively (Figure 4A). Then they were fused to produce fragment UP1-P_1503_PE-DN1 (Figure 4B) and transformed WT strain. Screened by erythromycin, the P_1503_PE-substituted *ireB* gene-deletion intermediate strain *ireB*^Δ^ PPE has been obtained (Figure 4C). The intermediate strains were confirmed to be resistant to erythromycin and sensitive to 0.05% *p*-Cl-phe (a mid-value concentration between MICs for WT and gP_1503_PE) (Figure 4D). Then an intermediate *ireB*^Δ^ PPE strain was used for the second transformation. The upstream and downstream sequences of *ireB* gene, this time named UP2 and DN2 to distinguish from the sequences for the first transformation, were amplified (Figure 4E) and fused as UP2-DN2 fragments (Figure 4F). The UP2-DN2 fragment was transformed into the *ireB*^Δ^ PPE strain, and plated on TSAS containing 0.05% *p*-Cl-phe for counter-selection. A total of 100 colonies were randomly picked and tested using PCR. The whole experiment was repeated three times. Each time, of the *p*-Cl-phe-resistant colonies, 100% harbored the corrected genetic deletion (Figure 4G), indicating that P_1503_-*mPheS* is 100% efficient as a CSM for *S. suis* counter-selection.

### Two-Step Strategy for Markerless Genetic Manipulation of *Streptococcus suis* Using P_1503_-*mPheS* as a Counterselectable Marker

**FIGURE 5:**
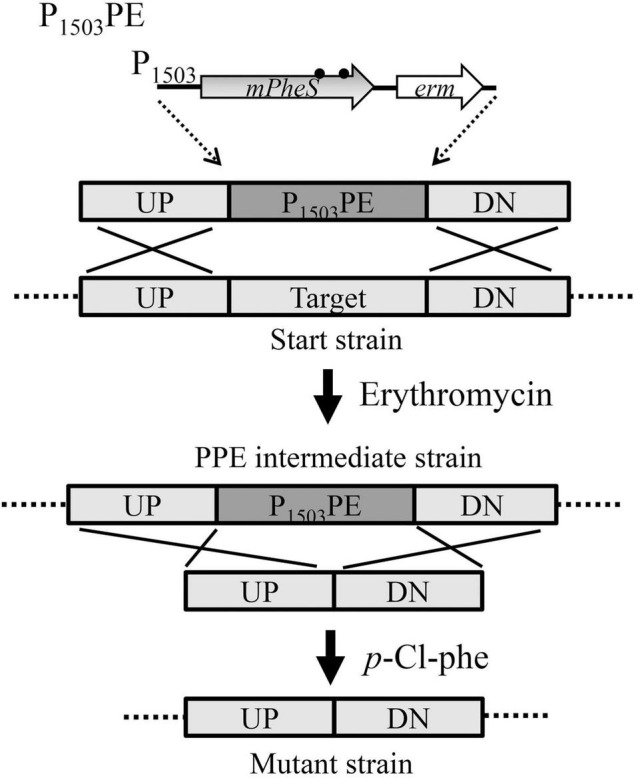
Diagram for the two-step markerless gene-deletion strategy developed for *S. suis* in this study, using *erm* as a positive-selection marker and P_1503_-*mPheS* as a CSM.

The 100% counter-selection efficiency of P_1503_-*mPheS* provides an intermediate strain that can be effectively removed using *p*-Cl-phe as a counterselective agent, thereby shaping a two-step insertion and excision strategy for markerless genetic manipulation of *S. suis*. As shown in the example of *ireB* gene-deletion and summarized in Figure 5, this strategy is clone-independent, and only two fused fragments and two transformations were needed. First, the upstream and downstream sequences of the mutation site were fused with P_1503_PE marker to yield the UP-P_1503_PE-DN fragment for the first transformation, which was selected by the introduced positive-selection marker *erm*, yielding a P_1503_PE-containing intermediate strain. Second, the upstream and downstream sequences of the mutation site are either directly fused together (for gene-deletion) or fused with a gene (such as gene of a fluorescent protein for gene-fusion), or fused with a mutated gene (for gene-mutation), to yield the second fragment for the second transformation, which was counter-selected by *p*-Cl-phe.

## Discussion

### P_1503_-*mPheS* Is the Most Efficient Counterselectable Marker for *Streptococcus suis* up to Now

Except for the above *ireB* gene-deletion, we have also applied the strategy to markerless construct *gfp*- or *rfp*-fusion strains (data not shown). In any case, the efficiency of the counter-selection is 100%, indicating that it is repeatable. This extremely high efficiency is not totally unexpected, as the mPheS-based CSM has been revealed as 100% efficient for counter-selection in *S. mutans* (Xie et al., 2011; Zhang et al., 2017). The high efficiency is further guaranteed by the wide difference between the MICs of *p*-Cl-phe to the strains with and without P_1503_-*mPheS*, which are 0.01 and >0.15%, respectively. Here in this study, we used a mid-value concentration (0.05%) between two MICs, there is still room to increase to ensure the 100% efficiency in future applications.

In contrast, the *sacB* gene and *yeoB* gene reported for *S. suis* is less efficient. When the *sacB* gene was introduced as a CSM for *S. suis*, the efficiency was not mentioned in that report (Zhang et al., 2019). We followed the report to use the native *sacB* gene and its promoter from *B. subtilis* for *S. suis* counter-selection, however, no target clone was obtained after screening several hundred colonies, suggesting a pretty low efficiency for counter-selection using this native *B. subtilis sacB* and promoter. We then adapted *sacB* gene codon for *S. suis* and utilized P_1503_ to drive the codon-adapted *sacB* gene expression. The efficiency was significantly increased and made *sacB* gene suitable for *S. suis* counter-selection. But in most cases, the counter-selection efficiency is about 50%. It is not surprising, as the MICs of sucrose for strains with and without P_1503_-*sacB* were around 7 and 10%, respectively (our unpublished data). This small difference between the two MICs makes counter-selection escape easy to happen, thus limiting the counter-selection efficiency. For the *yeoB* system, induction of the toxin only partially inhibits the growth of *yeoB*-containing strain, it is not surprising that the final culture needs to be enriched for several generations in presence of the inducer, but still, the efficiency could not reach 100% (Zheng et al., 2021). All in all, the P_1503_-*mPheS* developed in this study is the most efficient CSM for *S. suis* up to now.

### The Two-Step Markerless Genetic Manipulation Strategy Developed in This Study Is Time-Saving, Convenient, and Has Great Potential to Be Widely Used in *Streptococcus suis* Strains From Different Serotypes

Though the mPheS-based markerless gene manipulation strategy has been developed in several bacteria, the double-substituted mPheS-based CSM was newly developed and lacked extensive verification in other species. Therefore, it is still necessary to verify its efficacy in *S. suis* and determine many details for *S. suis*, such as the *pheS* gene itself, mutations to be introduced, appropriate selection concentration of *p*-Cl-phe, and especially the strong promoter specific for *S. suis*. All of these need to be carefully set up in the background of *S. suis*. Here in this study, we identified the *wtPheS* gene from *S. suis*. After the introduction of mutations and different promoters, we investigated the potential of these mPheS-based CSM for markerless gene manipulation in *S. suis*, either carried by plasmid or integrated into the genome. Finally, the P_1503_-*mPheS* was proved to be highly effective under 0.01% (or 0.5 mM) *p*-Cl-phe, and used to develop an efficient two-step strategy for markerless gene manipulation of *S. suis*. With this strategy, markerless gene manipulation of *S. suis* is time-saving and takes about 1 week, moreover, no unwanted recombination has been observed so far.

In consideration of convenience, mPheS-based CSM is also prior to the SacB- or YoeB-based CSM, as the counter-selection agent *p*-Cl-phe only needs to be added directly into the medium before autoclaving. In contrast, the sucrose needs to be sterilized by filtration separately and added after autoclaving, while the *yoeB* system needs several generations of enrichment with an inducer.

As both the *wtPheS* gene and P_1503_ are highly conserved in *S. suis*, the strategy developed in this study has great potential to be widely used in different *S. suis* strains. This strategy requires an efficient transformation method, which is not a problem because Zhu et al. (2019) recently developed the convenient peptide-induced transformation method for *S. suis* strains from different serotypes.

### Limitation and Optional Solution

Though the two-step strategy is very efficient, it has limitations. It needs two rounds of transformation. This can be problematic if the transformability of the strain is negatively affected after the first transformation step. To address this problem in *S. mutans*, Zhang et al. (2017) developed a next-generation counterselection cassette by introducing a short repeat sequence in the fused DNA fragment for the first transformation step. After obtaining the intermediate strain with positive selection, the strain was passaged several generations in absence of the selective agent. During the passages, the repeat sequence will mediate an *in vivo* recombination to remove the marker, and the markerless strains were then counter-selected by *p*-Cl-phe, thus skipping the second transformation step (Zhang et al., 2017).

We practiced this repeat sequence-mediated one-step strategy in *S. suis* mediated by a 200-bp repeat. Guaranteed by the high efficacy of counter-selection by P_1503_-*mPheS*, we successfully got the target markerless gene mutation strain with one transformation (data now shown). However, it was much trickier to fuse one more fragment by overlap extension PCR. We prefer to use the two-step strategy for the first choice, and when it is problematic, use the repeat sequence-mediated one-step strategy as an option.

In summary, this study supplies a rapid and efficient tool for sophisticated genetic analysis of *S. suis*. Hopefully, it will accelerate the physiological and pathological studies of this important zoonotic pathogen.

## Data Availability Statement

The data presented in the study are deposited in the GenBank repository, accession number ON184273.

## Author Contributions

YZ, GG, DW, GL, PC, and LW performed the experiments and data analysis. YZ and SL wrote the manuscript. All authors contributed to the article and approved the submitted version.

## Conflict of Interest

The authors declare that the research was conducted in the absence of any commercial or financial relationships that could be construed as a potential conflict of interest.

## Publisher’s Note

All claims expressed in this article are solely those of the authors and do not necessarily represent those of their affiliated organizations, or those of the publisher, the editors and the reviewers. Any product that may be evaluated in this article, or claim that may be made by its manufacturer, is not guaranteed or endorsed by the publisher.
